# Estimating the Prevalence of Opioid Diversion by “Doctor Shoppers” in the United States

**DOI:** 10.1371/journal.pone.0069241

**Published:** 2013-07-17

**Authors:** Douglas C. McDonald, Kenneth E. Carlson

**Affiliations:** US Health Division, Abt Associates Inc., Cambridge, Massachusetts, United States of America; University of Louisville, United States of America

## Abstract

**Background:**

Abuse of prescription opioid analgesics is a serious threat to public health, resulting in rising numbers of overdose deaths and admissions to emergency departments and treatment facilities. Absent adequate patient information systems, “doctor shopping” patients can obtain multiple opioid prescriptions for nonmedical use from different unknowing physicians. Our study estimates the prevalence of doctor shopping in the US and the amounts and types of opioids involved.

**Methods and Findings:**

The sample included records for 146.1 million opioid prescriptions dispensed during 2008 by 76% of US retail pharmacies. Prescriptions were linked to unique patients and weighted to estimate all prescriptions and patients in the nation. Finite mixture models were used to estimate different latent patient populations having different patterns of using prescribers. On average, patients in the extreme outlying population (0.7% of purchasers), presumed to be doctor shoppers, obtained 32 opioid prescriptions from 10 different prescribers. They bought 1.9% of all opioid prescriptions, constituting 4% of weighed amounts dispensed.

**Conclusions:**

Our data did not provide information to make a clinical diagnosis of individuals. Very few of these patients can be classified with certainty as diverting drugs for nonmedical purposes. However, even patients with legitimate medical need for opioids who use large numbers of prescribers may signal dangerously uncoordinated care. To close the information gap that makes doctor shopping and uncoordinated care possible, states have created prescription drug monitoring programs to collect records of scheduled drugs dispensed, but the majority of physicians do not access this information. To facilitate use by busy practitioners, most monitoring programs should improve access and response time, scan prescription data to flag suspicious purchasing patterns and alert physicians and pharmacists. Physicians could also prevent doctor shopping by adopting procedures to screen new patients for their risk of abuse and to monitor patients' adherence to prescribed treatments.

## Introduction

Since the mid-1990s, misuse of prescription drugs has emerged as a serious threat to public health in the US. Between 1995 and 2011, admissions to emergency departments for opioid misuse increased about ten-fold as did annual treatment admissions for opioid abuse [1], [2], [3], [4], [5]. Opioid overdose deaths rose from about 4,000 in 1999 to 16,651 in 2010, and are now twice as common as heroin and cocaine overdose deaths combined [6]. Drug poisoning deaths now match automobile accidents as the leading causes of unintentional death in the US [6], [7]. In 2009, an estimated one in seven US residents aged 12 and older admitted to past nonmedical use of opioids, meaning not as prescribed by an informed physician [8]. This is not just an American problem. Although the US accounts for about half of illicit opioid users worldwide, nonmedical use of prescription opioids is rising in many developed and developing countries while consumption of heroin and cocaine has been declining [9].

This epidemic of abuse arose in the wake of an enormous growth in prescribing opioids. While the US population increased only 16% between 1997 and 2011, the amounts of oxycodone sold by retail pharmacies increased by 1,259%. Amounts of hydrocodone, methadone, fentanyl and morphine sold by pharmacies increased by 356%, 1,099%, 711%, and 246%, respectively. Buprenorphine sales went from a mere 17 grams in 2002 to 1,639 kg in 2011 [10], [11].

This shift to a more expansive use of opioids occurred in the 1990s, when the professional norms and attitudes that prevailed for much of the Twentieth Century and which shaped prescribing behavior were eroded [12]. Following passage of the Harrison Act in 1914 and subsequent prosecutions of physicians who violated the law, physicians adopted a conservative attitude toward pain management, marked by a reluctance to use “narcotics” to treat non-cancer pain, especially chronic pain. Pain management was not part of the medical school curriculum. When opiates were used for pain relief, weaker ones–codeine and meperidine–were most commonly prescribed [13]. Reliable estimates of under-treatment of pain are not available but studies of disparities in treatment indicate that under-treatment was, and continues to be, widely prevalent in the U.S. [14]. In the 1990s, professional attitudes toward opioid treatment began to evolve rapidly. Influential voices proposed that pain be considered a “fifth vital sign”[15]. Some argued that under-treatment of pain was a public health concern [14], [16]. More potent opioids were developed and marketing campaigns by drug manufacturers reinforced a more expansive use of opioids [17]. Not only did the amounts of opioids sold increase, but sales of more potent opioids displaced weaker ones–codeine and meperidine. To inform medical providers, medical associations and boards began issuing practice guidelines that supported more flexible use of opioids, especially for patients suffering chronic non-cancer pain [18], [19], [20], [21], [22], [23], [24], [25], [26], [27].

Opioids can be diverted to illicit use by several means, including theft, smuggling, unlicensed Internet pharmacies, and other illegal channels, but in the US most are prescribed by physicians [28]. Some small proportion of physicians may knowingly prescribe opioids for patients they suspect of abusing them [29], but probably the more common event is for patients to obtain prescriptions for the same drugs from different unknowing physicians–a practice referred to as “doctor shopping.” Patients seeking drugs for misuse are able to do so by exploiting gaps and weaknesses in healthcare information systems. Lacking universally available healthcare records, physicians often have to rely on what new patients tell (or do not tell) them about the care they are receiving from others. By going to different pharmacies and paying cash, patients can avoid detection when buying opioids with overlapping prescriptions from different doctors. Such use of multiple prescribers is correlated with opioid abuse, injury and death [30], [31].

There are no national estimates of diversion by doctor shopping in the published literature. The few efforts to estimate its prevalence rely upon data limited to particular states or to larger samples of prescription records [32], [33], [34], [35]. These estimates range from 0.3%–12.8% of all patients purchasing opioids, depending upon location or study sample and the definition of doctor shopping used. Most studies select a number of different prescribers and/or pharmacies used by patients as a threshold indicating probable doctor shopping; some include the existence of overlapping prescriptions. Studies in Europe have used similar strategies for estimating pharmacy shopping or doctor shopping [36], [37]. Using state or region-level data to estimate national prevalence of doctor shopping is inappropriate, as there is wide geographic variation in the prevalence of opioid prescribing [12], [38], [39]. Claims data are of limited usefulness because they do not capture cash purchases. Whether doctor shoppers are few or many in proportion to all patients given opioids has implications for both clinical practice and drug control policy.

### Research Objective

This study estimates the prevalence in the US of patients who obtained prescriptions during 2008 from large numbers of different physicians–numbers that indicate probable doctor shopping. Further, we characterize this population of patients using the limited information available to us in prescription records: patients' ages, methods of payment (cash or insurance), numbers of opioid prescriptions dispensed, as well as amounts and types of opioids obtained.

## Methods

### Ethics Statement

All patient-identifying information in these prescription records had been removed for secondary analysis prior to our acquiring them. It was impossible for us either to re-identify patients or to gain their consent to examine their prescription records. Abt Associates' Institutional Review Board therefore waived the requirement for gaining patients' informed consent and approved the study.

### Data and Sample

Records were obtained under a time-limited license from IMS Health Inc. for 146.1 million prescriptions for opioids that contained buprenorphine, codeine/dihydrocodeine, fentanyl, hydrocodone, methadone, oxycodone, oxymorphone, propoxyphene, or tramadol dispensed during 2008 by approximately 37,000 retail pharmacies, including specialty pharmacy prescriptions and mail services. IMS Health Inc. employed a HIPAA-compliant procedure to link all prescriptions dispensed to the same patient, a feature of these data that enabled analysis of doctor shopping. Patients' last names, first names, dates of birth, gender, address, and payer ID were encrypted, and an algorithm was then used to produce a probabilistic match of all prescriptions based on these encrypted elements. These prescriptions were purchased by 48.4 million unique patients and were written by 907,782 unique prescribers–nearly all active prescribers of controlled drugs in the US that year. For a summary measure, opioid weights were converted to morphine equivalents [40]. The data provided to us replaced pharmacy and prescriber names with a unique anonymous identifier and reported their locations at the ZIP code level. Diagnostic or other clinical information was not available, which limited our ability to distinguish medically appropriate from suspicious patterns of opioid purchasing. (Having diagnostic information might improve the indicators of appropriate prescribing and of doctor shopping, but because doctor shoppers present false symptoms, and sometimes even falsified MRI reports, each prescription will be associated with a diagnosis that looks legitimate [34].)

These data are the property of IMS Health, but a license to use them can be obtained directly from IMS Health Government Solutions in Fairfax, Virginia for a fee.

### Deriving Nationwide Estimates from the Sample

The sample was limited to all opioids sold during 2008 by a large non-random sample of US retail pharmacies, which comprised 76% of all that were active that year. The proportion of pharmacies participating in the sample (the “coverage rate”) varied geographically and by store ownership. To estimate the numbers and amounts of all opioids sold in the US and the total number of opioid patients in the US population, we used information acquired by IMS about total amounts of each specific opioid product sold by pharmaceutical manufacturers and distributors to *all* independent retail pharmacies, chain pharmacies, and other pharmacies in each geographic area defined by the first three digits of their ZIP codes (ZIP3). This permitted computation of the ratio of the amounts dispensed by sampled pharmacies to amounts sold to all pharmacies of the same type in each ZIP3 area. The prescription data in the sample were weighted by the reciprocal of this ratio (separately for each ZIP3 area) to estimate total numbers of opioid prescriptions dispensed in 2008 by all retailers (223 million). This assumes that quantities per prescription were the same in the sampled and in non-reporting pharmacies.

To estimate the total number of unique patients in the US population from this sample, we counted for each unique patient the observed number of pharmacies used by that patient in the sample in each of three categories of pharmacies (chain, independent, and “other”). For patients who obtained prescriptions at more than one store, the probability that each store participated in data collection was assumed to be independent of whether the patient's other stores participated, and that the number of observed patients following each pattern of stores was exactly the number that would be expected given the coverage rate and the population number of actual purchases in each pattern. From this, we estimated the probability that each type of patient (defined by the number of stores where prescriptions were filled) would be observed in the data. The reciprocal of this probability was the sampling weight. Because these data covered most patients, nearly all weights were between 1.0 and 2.0, with most closer to 1.0. All analyses used weighted data.

This analysis was based on the 13.6 million observed (unweighted) patients who purchased at least one opioid prescription during the first 60 days of 2008. When weighted, these cases represented a national population of 19.0 million (weighted) patients. For each patient, we counted the number of different prescribers used during the last ten months of 2008. To compare the shoppers estimated from this subgroup with all opioid purchasers, we later recombined their records with those for all other opioid purchasers.

### Modeling Patients' Use of Different Prescribers

Patients who are prescribed opioids do not constitute a single population but rather a mixture of different populations that differ in their needs for pain relief and their illicit nonmedical use of opioids. Rather than defining a priori a prescribing/purchasing pattern thought to distinguish probable diversion from appropriate medical care, we used finite mixture models [41] to identify different populations (sometimes called “latent populations”) of opioid- purchasing patients who obtained prescriptions from differing numbers of prescribers. These models were used to fit a mixture of Poisson distributions with parameter λ where λ depended on the latent population, the age and gender of the patient, and whether any of the purchases involved cash. The fmm procedure in Stata statistical software was used for analysis [42].

For all patients in the sample who obtained at one or more opioid prescriptions during the first 60 days of 2008, we counted all other opioid prescriptions they purchased between March 1 and December 31, 2008 from any of the sampled pharmacies. We then modeled the count of distinct prescribers represented by observed prescriptions. This model consisted of a systematic part, which we called the patient's rate (λ) and a random part. Patients with identical λ can produce different counts. For example, one patient might make a purchase on December 30 and another similar patient might make a similar purchase on January 2, 2009. This completely arbitrary difference gives the first patient one more prescriber than the second patient has. We think of differences like this as a random part of the count.

Because the observed count included a random component, any estimate of the total number of extreme patients that applied a fixed criterion (for example, “More than 5 prescribers per year”) was guaranteed to misclassify some patients. Moreover, there was no reason to suppose either that the number of misclassifications was small or that the number of false positive errors was equal the number of false negative errors. On the contrary, when we examined the model developed below, we found that the number of such misclassifications was large, and there was no point at which the number of false positive classification errors equals the number of false negative classification errors.

The simplest model of count data is the Poisson distribution. The Poisson distribution has only one parameter (λ) and results from three simple assumptions:

1. Over short time intervals, the probability of observing a new prescriber is approximately proportional to the duration of the interval.
2. Over very short time intervals, the probability of observing more than one new prescriber is approximately zero.
3. The probability of observing an event in one time interval is independent of events before that interval.

None of these assumptions holds perfectly for prescription data: few doctors are available on Sundays, for example, and patients may exhaust supplies before seeking new prescriptions. However, for most patients, the rate of new prescribers in our data was well below one per month, and at time scales of months, such violations of the Poisson assumptions did not noticeably affect the distribution.

We used maximum likelihood regression to allow the parameter of the distribution to vary with known patient characteristics (age, sex, and insurance coverage). Most importantly, however, we allowed the distribution to be a mixture of two (or more) processes with separate rates. A finite mixture model combines all these requirements. The assumption is that there are two or more kinds of purchasers. (Call them A, B, and so on.) The Poisson distribution has a single parameter, λ. We allowed λ to differ among patients, so the model was 
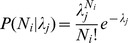
where 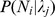 was the probability that the i^th^ patient received prescriptions from N different physicians during a ten-month period conditional on the rate parameter for new doctors being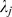. Let λ_A_, λ_B_, and λ_C_ be the respective parameters of groups A, B, and C. The probability density function of the observed number of different prescribers was given by 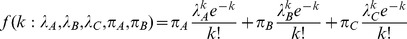 where π_A_ and π_B_, are the respective probabilities of membership in latent populations A and B, and π_C_ = 1−π_A_−π_B_.

In this model, the parameters depended on the patient's age, gender, and mode of payment. With 19 million observations, there was little danger of over-fitting, so we fit separate models for adults (anyone over the age of 14 years) and children, and for cash and non-cash (private insurance or Medicaid) payments. We treated children as a special case because they were the patients but not the purchasers. (Only 2.6% of the patients were children.) Therefore, we expected the functional forms relating their demographic characteristics to outcomes to be qualitatively different from those for adults. By similar logic, we fit separate models for anyone who made at least one cash purchase. There were four models, corresponding to the four age x payment combinations. For children, the model was 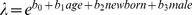


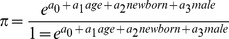



The adult model was almost the same: 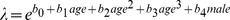


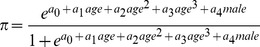



These models demand large sample sizes, sharp distinctions between types, and reasonable confidence in the distribution family being fit. The prescription data perfectly fulfilled the first two requirements. The shape of the distribution was an empirical question that we address below.

In addition to the rate parameter λ, the models estimated probabilities of each patient's being a member of each specified latent populations based solely upon information about patient's age, gender, and method of payment. We referred to this as the “prior” probability. Combining the prior probabilities with the observed number of different prescribers allowed us to estimate a “posterior” probability, which we used later to count and characterize the patients in each population.

## Results

**Figure 1:**
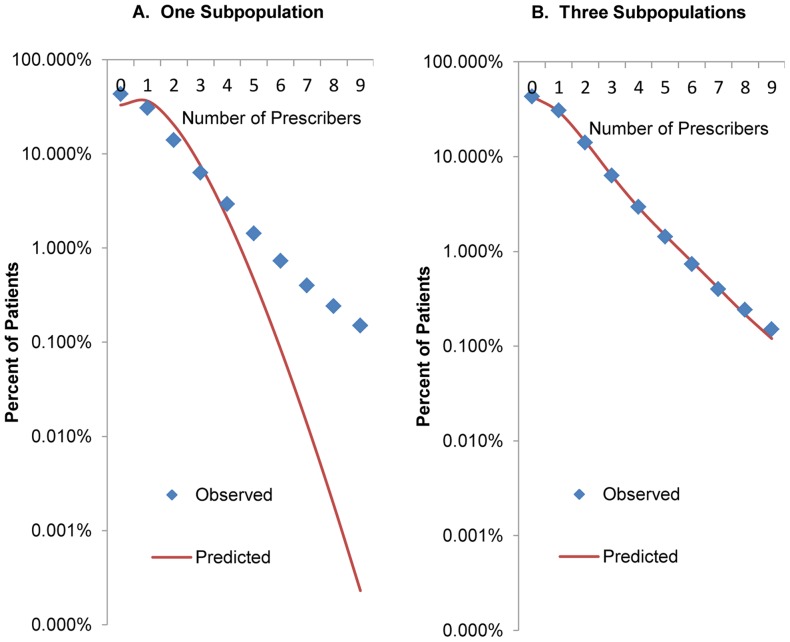
Observed and Predicted Numbers of Different Prescribers, Assuming One and Three Populations of Opioid Purchasers. Panel A illustrates that the number of different prescribers predicted using a Poisson model with only a single population shows that the assumption of a single patient population underestimates the number of different prescribers actually observed. Prediction based on the assumption that the population is a mixture of three different populations having distinct distributions of number of different prescribers fits the observed data very closely (Panel B). *Source*: Computed from LRx Data, 2008 obtained from IMS Health, Incorporated.

Among patients who were active opioid purchasers during the first two months of 2008, 43% had no further activity during the calendar year. Of those with some activity, most saw only one prescriber (31%) or two (14%). Three percent obtained prescriptions from 5–9 prescribers, 0.35% from 10–19, and 0.04% from 20 or more. Comparing the observed rates to the rates predicted using a Poisson model with only a single population shows that the assumption of a single patient population was not consistent with the data. The “observed” points in both panels of Figure 1 represents the fractions of patients who saw no physicians, or exactly one, or exactly two, etc. during the last ten months of 2008. The figure uses a logrithmic scale because after two doctors, the percentages of patients were very small.

The single-population model did not match the observed distribution: it systematically underestimated the number of patients with four doctors, and performed progressively worse as the number of doctors increased. The lack of fit for small numbers of doctors is obscured by the log scale, but the single-population model had too few zeros and too many twos. Of the 19 million weighted patients on whom this model was fit, the single-population model predicted that about 19,000 would have more than five physicians. In fact, 359,000 had more than five.

Testing models with two or more latent populations, we determined that a model representing a mixture of three different latent populations fit the observed numbers of different prescribers well, and that more populations did not further improve the fit. The predicted distribution closely approximated the actual observed distribution (Figure 1, right panel). The three distinct populations are designated sets 1, 2, or 3. Table 1 shows the parameters resulting from our estimation. The two columns on the left show estimated mean numbers of different prescribers for children and adults in each latent population, distinguishing those who made at least one cash purchase from those who relied entirely on insurance (including Medicaid). The two columns on the right show the prior probabilities, that is, the initially estimated probability that a patient came from these populations based only upon the information in the independent variables (the patient's age, gender, and method of payment). The computed probabilities and numbers of prescribers are specific to single years of age. Table 1 shows the average of these estimates across patients in each group. For example, 88.3% of children under 15 who had at least one cash purchase are estimated to come from population 1.

**Table 1:** Mean Number of Prescribers for Three Populations of Patients, by Patient Age and Payment Method.

		Mean Number of Different Prescribers		Prior Probability of Group Membership	
		Age <15	Age 15+	Age <15	Age 15+
**Population 1**	Cash	0.21	0.86	0.883	0.819
	Insured	0.08	0.71	0.832	0.844
**Population 2**	Cash	1.96	3.90	0.112	0.170
	Insured	0.81	2.75	0.159	0.150
**Population 3- Extreme**	Cash	9.08	14.86	0.005	0.011
	Insured	4.91	7.62	0.009	0.006

*Note*: Includes count of different prescribers during last ten months of 2008. “Insured” includes patients who used only private insurance or Medicaid for all purchases. “Cash” includes patients who ever paid cash for any purchase, even if they used insurance for others.

*Source*: Computed from LRx Data, 2008 obtained from IMS Health, Incorporated.

Population 1 consisted of patients who averaged less than one new prescriber during the ten-month period. Most patients were in this population: the probability of being in Population 1 was between 81.9% (adults who paid cash) and 88.3% (children with cash payments). Although all had gotten at least one prescription during the first two months 2008, most had no reason to obtain another during the following ten months. The second population of patients obtained opioid prescriptions during the ten-month period from an average of between one prescriber (for insured children) and four prescribers (for adults who paid cash), and comprised 11%–17% of patients in their respective age x payment strata. In the third population, labeled “extreme,” cash-paying adults obtained opioid prescriptions from an average of 15 different prescribers in ten months. Approximately one percent of all cash-paying adults came from this population, as did between one-half and one percent of those in other strata. All patients combined in this third population got prescriptions from an average of 10.4 different prescribers during these 10 months.

**Figure 2:**
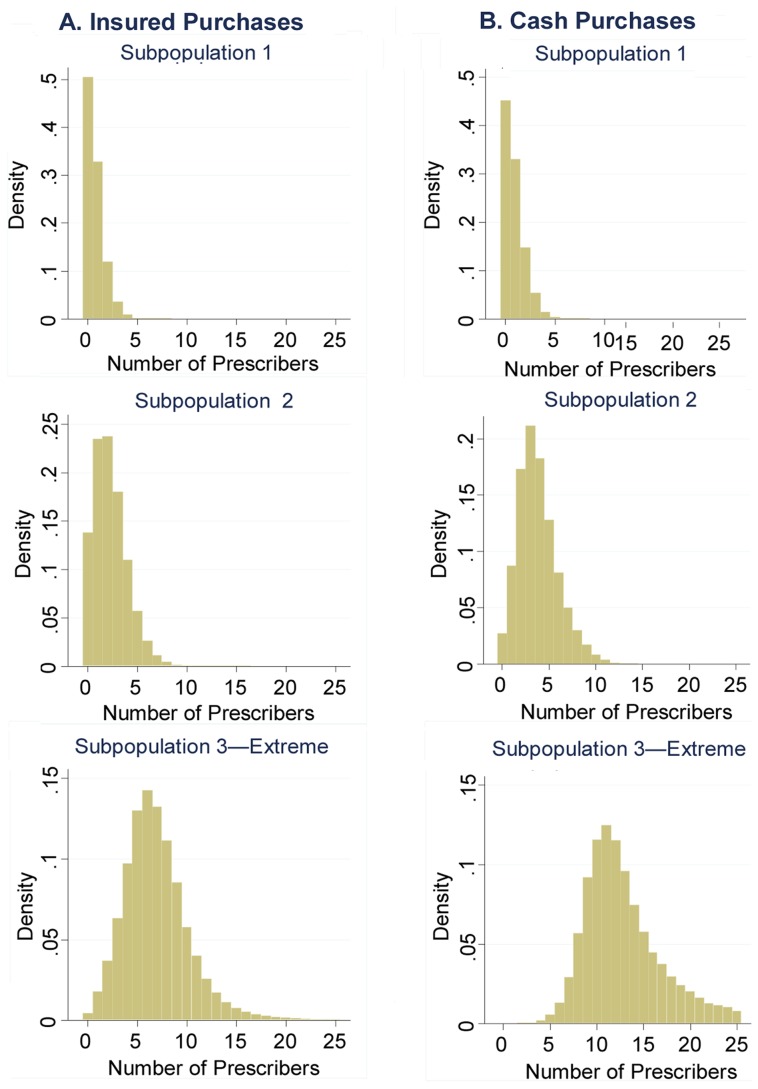
Observed Distributions of Number of Opioid Prescribers for Three Populations of Patients, by Payment Method. Figure shows the distribution of actual rates of using different prescribers for patients predicted to be in each of the latent populations, showing patients who purchased all prescriptions with insurance separately from those who paid cash for at least one prescription. The distributions of observed rates conformed quite closely to the predicted distributions for the different populations, showing random variation around the mean rate in each. The vertical axis is scaled so that the area of each graph equals 1. *Source*: Computed from LRx Data, 2008 obtained from IMS Health, Incorporated.

Figure 2 shows the distribution of actual rates of using different prescribers for patients predicted to be in each of the latent populations, showing cash-paying patients separately from those who used only insurance. The distributions of observed rates conformed quite closely to the predicted distributions for the different populations, showing random variation around the mean rate in each. For patients in population 1, zero was the most frequent number of observed different prescribers. As described above, patients were selected for this analysis if they received at least one prescription from at least one prescriber during the first 60 days of 2008, and the analysis examined prescriptions obtained after that period. One would therefore expect to see many zeros for this population. However, even for the third population, zero was a possible observation. For example, the mean number of prescribers for nine-year-old boys in population 3 who made no cash purchases was 5.67 different prescribers per 10-month period. In a Poisson distribution with mean 5.67, about one case out of every 290 will have zero observed events. Since we had 8,352 nine-year-old boys with zero different prescribers, we estimated that a few of them (19) came from population 3.

We used the estimated prior probabilities in Table 1 plus the actual number of different prescribers observed to reconstitute a synthetic estimate of the population of extreme users. We computed the posterior probabilities for each patient, i.e., the probabilities that the patient came from each of the three groups given both the independent variables and the observed number of different prescribers. Each patient had three such posterior probabilities, one for each population. We can think of the probabilities as the fractions of similarly situated patients who came from each group. For example, we had 372 actual observations (representing 437 weighted patients) of women born in 1972 who never made a cash purchase and who saw exactly 7 different prescribers during the ten month period. The posterior probability of coming from population 3 for each member of this group was 0.47, so we estimated that this group contained 205 (437×0.47) members of population 3.

### Age and Numbers of Different Prescribers

**Figure 3:**
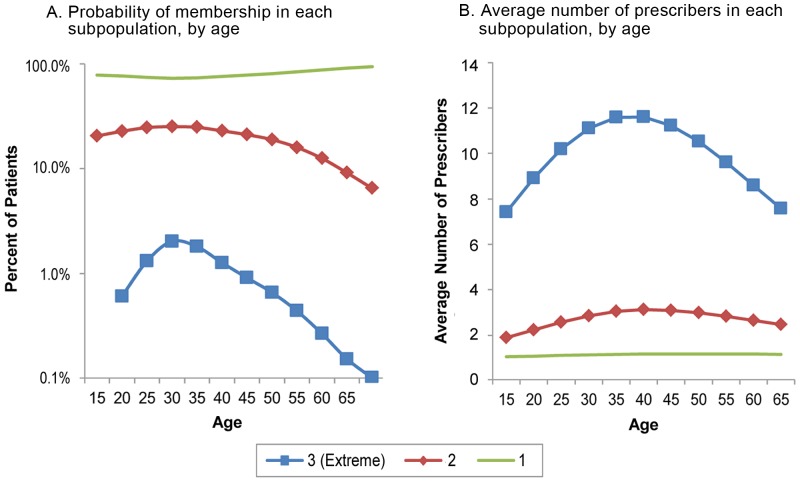
Probability of Membership and Average Number of Different Prescribers in 3 Latent Populations, by Age. The predicted probability of being in each of the three latent patient populations varied according to patients' age (Panel A), with the probability of being in the doctor shopping population (3- extreme) highest among patients in their thirties. Patients in this extreme population (presumed doctor shoppers) obtained prescriptions from many more different prescribers than patients in other populations (Panel B). The average number of different prescribers used by patients predicted to be in the doctor shopper population (3) peaked in the 30–45 age range. *Source*: Computed from LRx Data, 2008 obtained from IMS Health, Incorporated.

At every age, a substantial majority of patients (about three quarters of those between the ages of 15 and 50, and more for older patients) were in the category we labeled as coming from population 1–that is, for whom a single prescriber at most was typical (Figure 3). After age 60, the majority grew to about 90 percent. At every age, opioid patients in this group saw an average of about one prescriber over the 10 months we observed. Patients in Population 2 saw an average of two to three prescribers, increasing only slightly with age. The number of patients in this group actually fell slightly after age 50 because they were less likely to get opioid prescriptions from multiple physicians.

The characteristics of Population 3 varied substantially with age: patients in their mid to late twenties were ten times as likely to fit the prescription patterns of extreme users as patients twice their age. Among Population 3, the average number of physicians increased with age until approximately age 40, and then declined among older patients.

### Estimated Number of Doctor Shoppers in US and Amounts Diverted

We applied our composite probability distribution to each patient to calculate the probability that the patient was a member of the “extreme” group. That is, we multiplied the size of each stratum of patients by its posterior probability of population 3 membership to estimate the total number of probable shoppers in the United States. Summing these probabilities, we estimated that of the 19 million patients in the US who purchased opioids in the first 60 days of 2008, 135,000 (0.7%) were members of this extreme population (Table 2).

**Table 2:** Active Opioid Patients and Estimated Number of Doctor Shoppers, by Age and Payment Method, 2008.

	Patients Filling at Least One Opioid Prescription During the First Two Months of 2008			Estimated Number of Doctor Shoppers During 2008		
	Age <15	Age 15+	Total	Age <15	Age 15+	Total
**Cash**	74,714	4,750,620	4,825,334	357	53,153	53,510
**Insured**	419,160	13,772,465	14,191,625	3,614	78,297	81,911
**Total**	493,874	18,523,085	19,016,959	3,971	131,450	135,421

*Source*: Computed from LRx Data, 2008 obtained from IMS Health, Incorporated.

Although only a small fraction of active patients, members of this extreme population obtained an estimated 1.9% (4.3 million) of all 223 million opioid prescriptions dispensed during 2008, and 2.8% of all oxycodone prescriptions (Table 3). They purchased an average of 32 opioid prescriptions that year. When we accounted for the quantity of drugs prescribed, their share of the market was even larger: an estimated 4.0% of the total amounts of these drugs dispensed that year, or about 11.1 million grams. This was equivalent to approximately 5.4 million grams of morphine. This would have provided an average of 109 morphine equivalent milligrams per patient in this extreme group for every day in 2008.

**Table 3:** Extreme Patients' Purchases as Estimated Share of Total Opioid Market, 2008.

	Prescriptions		Amounts (grams)	
	Number of prescriptions	Share of all prescriptions dispensed	Number of grams	Share of all grams dispensed
Buprenorphine	62,000	1.9%	33,000	4.2%
Codeine	179,000	1.2%	844,000	1.8%
Fentanyl	7,000	1.2%	200	4.2%
Hydrocodone	2,132,000	1.8%	1,799,000	3.9%
Methadone	1,000	1.0%	7,000	3.6%
Oxycodone	1,246,000	2.8%	2,424,000	5.3%
Oxymorphone	12,000	2.3%	41,000	6.2%
Propoxyphene	205,000	1.0%	2,089,000	2.1%
Tramadol	441,000	2.0%	3,892,000	4.7%
All Prescriptions	4,285,000	1.9%	11,129,200	4.0%
All Prescriptions, morphine equivalents			5,385,000	

*Notes*: Total opioid market refers to prescriptions filled for all patients who made at least one purchase between January 1 and December 31, 2008.

*Sources*: Computed from LRx Data, 2008 obtained from IMS Health, Incorporated. All rights reserved.

The risk of abuse, as measured by prescriptions obtained by doctor shoppers as a percent of all prescriptions dispensed, was highest for oxycodone–2.8% (Table 3). This is consistent with findings in studies of smaller samples [43]. Oxymorphone was slightly less likely to be purchased by doctor shoppers (2.3%), followed by tramadol (2.0%), buprenorphine (1.9%) and hydrocodone (1.8%). Risks were lowest for codeine/dihydrocodeine (1.2% of all prescriptions dispensed of this drug), fentanyl (1.2%), methadone (1.0%), and propoxyphene (1.0%).

Because we did not have any clinical information other than these patients' purchases, we could not empirically prove that opioid use by patients in this extreme population was not medically appropriate. However, we classified these patients as highly suspect on the basis of this fact alone and assumed that they were doctor shoppers. Several other findings reinforce this assumption. They obtained an estimated average of 32 opioid prescriptions from an average of 10 different physicians in 2008. They purchased enough opioids to provide a very high daily dosage (109 morphine equivalent milligrams) for all 365 days that year. The probability of being in this most extreme population was highest among persons 26–35 years olds (Figure 3), the same age range having the highest prevalence of self-reported nonmedical use of prescription drugs during the past month [44].

### Limitations

There are several possible sources of error in our estimates. Prescriptions purchased by same individuals may not have been matched or linked correctly. Patients using aliases were not matched. The use of aliases is probably infrequent in half of the states that require strong identification before dispensing opioids [45]; frequency of use in other states is unknown. The sample of pharmacies reporting data, although very large, was not random, and therefore had systematic bias. Independent pharmacies were underrepresented and there were a few small, rural areas where no pharmacies contributed data. We had no estimates for these areas. In a few others, the estimated sample coverage was less than 2% of purchases. We limited the sampling weights assigned to purchases in these areas to be no greater than 50.0 to avoid having a few extremely influential observations. This caused us to undercount purchases.

The data for this study were limited to drugs dispensed by retail pharmacies and not by clinics, hospitals, or by practitioners directly. (Drugs *prescribed* by physicians in hospitals and clinics and dispensed by retail pharmacies were included, however.) Doctor shopping may be therefore be underestimated slightly by the absence of data from hospitals and from pain clinics that exist in some states and that have come under increased scrutiny by diversion control agencies [46]. Most opioids were dispensed by retail pharmacies in 2008. According to the Drug Enforcement Administration, 86% of all opioids, measured in grams, dispensed that year were dispensed by retail pharmacies, 7.3% by hospitals, 5.7% by narcotic treatment facilities (methadone maintenance facilities), and less than 1% by practitioners, including those working in clinics [11]. If opioids dispensed by pain clinics and hospitals were counted, our estimate of doctor shoppers would probably increase slightly. Although retail pharmacies dispensed most opioids in the US during 2008, doctor shoppers may have disproportionately favored these unmonitored outlets. Inclusion of data from narcotic treatment facilities would probably not affect the estimate of shoppers, as doctor shopping is indicated by the numbers of *different* prescribers, rather than the total number of prescriptions or amounts of drugs dispensed. Reducing by 1 the count of prescribers/dispensers used by treatment center clients who were doctor shopping would have slightly changed the probability estimate of being in the extreme/shopper population for every patient, but the model assumes that both shoppers and non-shoppers see randomly varying numbers of prescribers, and differences like this are well within the range of random variation for doctor shoppers.

Some undetermined number of patients obtained prescriptions from different prescribers who worked in the same group practice and who would have been fully informed about their patients' histories. This also falls within the range of random variation allowed by the model.

Our estimates relied on the assumption that patients' rates of using new physicians to obtain prescriptions were Poisson distributed. Possible violations of the Poisson assumption may have come from two sources: (1) a patient might have come away from a primary care contact with a short-term opioid prescription and a referral to a specialist, who subsequently wrote a new prescription, and (2) the limited demographic variables available for this analysis did not completely account for patient variability in rates of prescriber utilization. The effect of both of these would have been to increase the variance of the distribution of number of prescribers. Any such increase in variance would leave the estimated number of shoppers smaller than that we estimate here. Our estimates should therefore be considered approximate upper bounds.

## Discussion

We estimate that a small outlying population of approximately one of every 143 patients who purchased opioids from retail pharmacies in 2008 obtained these prescriptions from a suspiciously large number of different prescribers. Patients in this population got an average of 32 prescriptions from an average of 10 different physicians during the last ten months of that year; those who bought at least one of these prescriptions with cash–arguably an indicator of covert doctor shopping–got prescriptions from an average of 15 different physicians. Those whose purchases were all covered by insurers got prescriptions from an average of eight different physicians, still a suspiciously large number. These are averages, and some patients saw over 200 different prescribers that year. Patients in this group purchased a disproportionate share of all opioid prescriptions (approximately 1 of every 50) sold that year and a disproportionate amount (1 of every 25 grams sold).

Because opioid diversion by doctor shopping is a covert activity, we only assign probabilities of shopping. We cannot identify individual shoppers from prescription data alone. Prescription data do not reveal the reasons why patients obtained prescriptions from so many different prescribers. Nor do they reveal the prescribers' reasons for writing the prescription or their understanding of the patients' motives. Numbers of different prescribers is therefore a signal of the probability of being a doctor shopper rather than an identifier. Other indicators, including overlapping prescriptions, dose escalation, and use of different pharmacies have been correlated with abuse or misuse in some studies [47], [48]. Additional analyses using such characteristics may afford more accurate assignment of patients to the appropriate latent populations.

Even though our indicator of doctor shopping–the numbers of different prescribers used during 2008–is probabilistic rather than determinative, the patterns observed in this extreme population of about 135,000 patients are above the thresholds of 6+ different prescribers in a year that members of an expert panel agreed is a signal warranting closer evaluation of patients' drug use [47]. As shown above, patients in this outlier population presumed to be doctor shoppers obtained prescriptions from an average of 10 different prescribers in 2008. The amounts of opioids purchased by patients in this extreme population are another strong signal of doctor shopping–an estimated average of 109 morphine equivalent milligrams per patient per day for the entire year. Even if these patients did not divert drugs for illicit purposes, obtaining opioids from such large numbers of prescribers may signal dangerously uncoordinated care, which poses the same risks to health as intentional abuse/nonmedical use.

Our approach involved estimating the numbers of different latent populations (three), the numbers of patients in each of these populations, and the probabilities that each patient would be in each of the three populations. This probabilistic identification did not rely upon *classifying* each patient into one of the three latent populations. Other studies have estimated the prevalence of doctor shoppers by classifying individual patients in a sample using of one or more characteristics. None has sought to estimate prevalence at the national level. These various estimates therefore differ from ours. For example, an analysis of Massachusetts' prescription drug monitoring program (PMP) records for Schedule II opioids prescribed during 2006 estimates that approximately 9,000 patients were engaged in “questionable activity” indicative of doctor shopping, defined as using ≥3 different prescribers and ≥3 pharmacies that year [34]. These constituted 1.6% of all patients obtaining Schedule II opioids that year, but they purchased a disproportionate amount of Schedule II opioids: 112,381 prescriptions (7.7% of all dispensed), and 7.6 million (8.5%) dosage units. Using a criterion of ≥5 prescribers and ≥5 pharmacies, the estimates shrank to 1,149 (0.2%) patients, 22,000 (1.5%) prescriptions, and 1.2 million (1.4%) dosage units.

An analysis of California's PMP records for prescribed Schedule II, II, and IV opioids defined a “doctor shopping episode” as a patient receiving prescriptions for the same medication from to two or more different prescribers and filled by two or more different pharmacies within any 30-day period during 2007 [49]. Doctor shoppers so defined obtained an estimated 12.8% of all prescriptions in the state for opioids during 2007. The number of such episodes was not reported, so we cannot compare it to our measure of total number of different prescribers used in a year. Having only one such episode in a year is an expansive definition that would include many legitimate transactions, such as refilling a prescription only once during the months, obtaining prescriptions from physicians in the same practice, etc.

A second study of California's PMP records by the same authors compared patients receiving opioids from two to five different prescribers during a one-year period to patients using only one prescriber [50]. This study found no evidence that patients in the former group were more likely to be abusing opioids than patients in the second group, suggesting that the threshold for suspected doctor shopping based on numbers of different prescribers should be higher than five. This is consistent with our estimates that patients having two to five different providers in a year have a higher probability of being in the second of the three latent populations and not in the outlier population of presumed doctor shoppers (Table 1). We estimate that this second population comprises 12 to 17% of all opioid purchasers nationwide.

A pair of studies developed estimates of doctor shopping using a large sample of pharmacy prescription records obtained by IMS Health during 2008 [33], [35]. This sample was similar to the one we used but some selection rules differed, patients were tracked for 18 months, and the authors did not weight the data to estimate nationwide prevalence. Similar to the finding reported for California, 13.1% of opioid patients obtained at least two overlapping prescriptions from different prescribers during at least one 30 days period [33]. Doctor shopping was defined as ≥2 prescriptions by different doctors with ≥1 day overlap and filled at ≥3 different pharmacies, and 0.3% of patients who purchased opioids in 2008 met this criterion [35]. This estimate was less than our estimate of 0.7%. Again, our approach was to model multiple populations rather than to classify patients using one or more threshold criteria and then to derive estimates for the entire nation. Some of the difference in estimates may stem from difference in these authors' sample and our weighted national sample, as well as from the use of other criteria (overlapping prescriptions and numbers of pharmacies). As stated above, our estimates should be considered as approximate upper bounds.

Prescription data were not obtained for analysis later than 2008, and we can only speculate about changes in the prevalence of doctor shopping since then. The amount of potent opioids sold by retail pharmacies has continued to increase. Between 2008 and 2011, the most recent year for which Drug Enforcement Administration data on sales are available, amounts of oxycodone sold increased 32%, hydrocodone 40%, methadone 17% and an equivalent increase for morphine, and amounts of buprenorphine nearly doubled (97%) [11]. As discussed in the introduction, various indicators of opioid misuse climbed higher year by year since the mid-1990s, more or less in tandem with rapid growth in the prescribing and sales of opioids. We do not know the precise relationships among changes in amounts prescribed, numbers of patients, and numbers of doctor shoppers. If these increases in amounts sold indicate correspondingly larger numbers of patients, and if doctor shoppers as a proportion of all patients is approximately the same as in 2008 (an estimated 1 in 143 patients), then doctor shopping may have been more prevalent in 2011 than it was in 2008.

Even in an environment of increasingly plentiful opioid prescribing, there exist approaches to preventing the small proportion of opioid patients who doctor shop to acquire drugs for misuse. One such approach is to close the information gap that allows doctor shopping to occur. Doctor shoppers are able to exploit physicians' difficulty in acquiring information about their patients' prescription histories. Lacking readily accessible information, they have had to rely on what their patients tell them. Beginning with New York in the early 1900s, several states passed laws requiring central monitoring of prescriptions for selected drugs, but these used paper systems (multiple prescription forms, typically) that may have been useful for investigators but which did nothing to inform physicians. In the 1990s, several states established computer-based prescription drug monitoring programs (PMPs) to collect electronic information from pharmacies about dispensed prescriptions of certain specified scheduled drugs–typically, all Schedule II drugs and various others. States passed laws requiring pharmacies to report these specified drugs to the PMP. Federal assistance for these programs became available in 2002 with the creation of the Prescription Drug Monitoring Program within the U.S. Department of Justice [51], which spurred the creation of state-government PMPs or enhancements of existing ones. In 2005, Congress passed the National All Schedules Prescription Electronic Reporting Act (NASPER), which provided additional funding through the U.S. Department of Health and Human Services to support further enhancements of state PMPs, including more expansive reporting and cross-state data sharing procedures [52], [53]. By 2012, programs had become operational in nearly all states. All programs have procedures to allow physicians to access the PMP database to obtain their patients' prescription histories. To date, however, most physicians have not incorporated into their work routines such data collection in advance of prescribing opioids. In 2010, only a small minority of prescribers had even registered to access these monitoring data–between 5 and 39%, depending upon the state [54]. Accessing the data is a cumbersome process in many states, which hinders integrating it into physicians' workflow. Only two states (Kentucky and W. Virginia) legally require prescribers to access prescription histories in the PMP databases before writing prescriptions for new patients for certain specified opioids, although 10 others require it if prescribers suspect the patient of abusing drugs [55]. Shoppers who operate in different states may evade detection by any single state's PMP, although cross-state data sharing agreements will help to ameliorate this deficiency. A number of pilot projects are being conducted in several states to test more effective and efficient integration of PMP data into patient records [54]. Going forward, the most promising path to preventing and detecting doctor shopping will be to integrate PMP data into patients electronic medical records; for physicians to make more inquiries to PMPs about their patients, especially those they suspect of drug seeking; and for PMP administrators to improve response speed and efficiency so that the barriers to routine acquisition of patients' prescription histories will become lower.

Another approach that does not specifically target doctor shopping but looks at misuse more generally is to tighten controls on opioids. This could be accomplished by the Drug Enforcement Administration's lowering the aggregate production quotas, which are set annually. This path has not been followed, however, as quotas for opioids have typically been raised over the years [56]. Another approach is to reclass particular opioids to more restrictive schedules, thereby narrowing physicians' discretion in prescribing. In late 2012, for example, the Food and Drug Administration, which controls the scheduling of prescription drugs, initiated consideration of moving hydrocodone, the most commonly prescribed opioid, from Schedule III to Schedule II [57]. In March of that year, the New York State Attorney General used state law to reclass this same drug to Schedule II [58]. One risk of “up-scheduling” hydrocodone products is that they may become less available to patients who have legitimate needs for pain relief. Tightening prescribing regulations for benzodiazepine agents in New York State during the late 1980s resulted in curtailing their legitimate use for chronically ill patients in need of the drugs for treatment [59].

There remain a number of open questions about opioid diversion by doctor shopping that are deserving of research attention. These include, among others:

- What proportion of the illicit market for opioids is supplied by doctor shopping as opposed to unethical prescribers or pharmacies, other forms of prescription fraud, smuggling, theft, pilfering, or unused pills left over from prescriptions written for legitimate medical reasons?
- Is doctor shopping more prevalent in some places than others, and if so, what accounts for these differences? Other studies find that abuse of both illicit and prescription drugs varies by region, so it is likely that diversion by doctor shopping also varies by geography [28], [60], [61].
- To what extent do doctor shoppers seek drugs for their own misuse as opposed to reselling them to others?
- Some doctor shoppers operate in rings that are organized and financed by leaders [62]; how much of the illicit market is supplied by these organized rings as opposed to solo shoppers?
- How much doctor shopping occurs across state borders and is thereby less visible to prescription monitoring programs in any one state? How effective are prescription monitoring programs in preventing doctor shopping?

Answers to these questions will advance our knowledge of opioid diversion practices and thereby inform the design of policies and programs to prevent and control diversion.

Ultimately, the front lines of defense against drug diversion by deceptive patients are manned by physicians and other healthcare providers who are authorized to prescribe scheduled drugs. Many of these providers are ill prepared. Pain management has not been traditionally been a part of the curriculum in medical schools and surveys of physicians have found their knowledge of pain management principles to be deficient [63], [64]. To remedy this, several organizations have issued practice guidelines designed to meet the demands for opioid use in pain management while mitigating the risks of misuse, abuse, addiction, and adverse events such as overdoses [21], [27]. Moreover, there exist a number of tools that they can use to maximize effective treatment while minimizing the risks, including addiction screening instruments to administer prior to prescribing, inquiries to PMPs to document patients' prescription histories, brief interventions, referral criteria, and various methods of monitoring adherence to treatment [65]. PMP administrators can also support prescribers proactively by scanning prescription data to identify patients who receive opioid prescriptions from large numbers of different physicians and then notifying the physicians of these findings. For such proactive notification to be cost-effective, data mining capabilities and secure electronic communication networks are necessary, which do not yet exist in many states but can be developed.
